# Novel Discharge Center for Transition of Care in Vulnerable Emergency Department Treat and Release Patients

**DOI:** 10.7759/cureus.34937

**Published:** 2023-02-13

**Authors:** Lisa O Iyeke, Bibi Razack, Mark Richman, Adam J Berman, Frederick Davis, Helena Willis, Marina Gizzi-Murphy, Stephen Guilherme, Sarah Johnson, Chinna Njoku, Genelle Ramjattan, Katarzyna Krol, Nancy Kwon

**Affiliations:** 1 Emergency Medicine, Long Island Jewish Medical Center, New Hyde Park, USA; 2 Medical Toxicology, Long Island Jewish Medical Center, New Hyde Park, USA

**Keywords:** transitions of care, operational metrics, discharge process, vulnerable patients, emergency medicine, emergency department

## Abstract

Introduction

The majority of emergency department (ED) patients are discharged following evaluation and treatment. Most patients are recommended to follow up with a primary care provider (PCP) or specialist. However, there is considerable variation between providers and EDs in discharge process practices that might facilitate such follow-up (e.g., simply discharging patients with follow-up physician names/contact information vs. making appointments for patients). Patients who do not follow up with their PCPs or specialists are more likely to be readmitted within 30 days than those who do. Furthermore, vulnerable patients have difficulty arranging transitional care appointments due to poor health literacy, inadequate insurance, appointment availability, and self-efficacy.

Our innovative ED discharge process utilizes an Emergency Department Discharge Center (EDDC) staffed by ED Care Coordinators and assists patients with scheduling post-discharge appointments to improve rates of follow-up with outpatient providers. This study describes the structure and activities of the EDDC, characterizes the EDDC patient population, and demonstrates the volume and specialties of appointments scheduled by EDDC Care Coordinators. The impact of the EDDC on operational metrics (72-hour returns, 30-day admissions, and length-of-stay [LOS]) and the impact of the EDDC on patient satisfaction are evaluated.

Methods

The Long Island Jewish Medical Center (LIJMC) EDDC is an intervention developed in July 2020 within a 583-bed urban hospital serving a racially, ethnically, and socio-economically diverse population, with many patients having limited access to healthcare. Data from the Emergency Medicine Service Line (EMSL), an ED Care Coordinator database, and manual chart review were collected from July 2020 to July 2021 to examine the impact of the EDDC on 72-hour returns, 30-day admissions, and Press Ganey's® "likelihood to recommend ED" score (a widely used patient satisfaction survey question). The EDDC pilot cohort was compared to non-EDDC discharged patients during the same period.

Results

In unadjusted analysis, EDDC patients were moderately less likely to return to the ED within 72 hours (5.3% vs. 6.5%; p = 0.0044) or be admitted within 30 days (3.4% vs. 4.2%). The program was particularly beneficial for uninsured and elderly patients. For both EDDC and non-EDDC patients, most revisits and 30-day admissions were for the same chief complaint as the index visit. The length-of-stay increased by ~10 minutes with no impact on satisfaction with ED visits. Musculoskeletal conditions (~20%) and specialties (~15%) were the most commonly represented. Approximately 10% of referrals were to obtain a PCP. Nearly 90% were to new providers or specialties. Most scheduled appointments occurred within a week.

Conclusion

This novel EDDC program, developed to facilitate outpatient follow-up for discharged ED patients, produced a modest but statistically significant difference in 72-hour returns and 30-day admissions for patients with EDDC-scheduled appointments vs. those referred to outpatient providers using the standard discharge process. ED LOS increased by ~10 minutes for EDDC vs. non-EDDC patients, with no difference in satisfaction. Future analyses will investigate impacts on 72-hour returns, 30-day admissions, LOS, and satisfaction after adjusting for characteristics such as age, insurance, having a PCP, and whether the scheduled appointment was attended.

## Introduction

Nationally, 77% of Emergency Department (ED) patients are discharged following evaluation and treatment [1,2]. Such "treat-and-release" ED patients are often given recommendations to follow up with a primary care provider (PCP) or specialist after discharge. Patients benefit from short-term follow-up from the ED with regard to managing chronic illnesses or providing care for acute conditions diagnosed in the ED. Access to a PCP and/or a regular health facility contributes greatly to health outcomes. Timely follow-up after discharge from the ED improves patient-centered outcomes. For instance, patients who follow up with a PCP after discharge from a hospital are 10 times less likely to be admitted within 30 days than patients who do not follow up [3]. However, patients may face numerous challenges in securing and actually attending post-discharge outpatient appointments, including poor insight into the value of close follow-up, a lack of insurance, and ineffective discharge planning (including not understanding discharge/follow-up instructions) [4]. Many patients seen in the ED lack a PCP or specialist, which makes it difficult for them to access healthcare in a timely manner [5]. Even when patients have a PCP or specialist, these providers are often unaware that their patients visited an ED. Scheduling timely appointments has become increasingly challenging, with a trend towards greater wait times for appointments that is unlikely to improve based on projections of physician supply and demand [6]. Patients with a PCP or specialist may still have difficulty arranging their own follow-up on account of lack of appointment availability, inadequate insurance, or poor health literacy, self-efficacy, comprehension, or adherence to their treatment plan [7,8].

In addition, discharge planning varies greatly across EDs, even within the same health system and geographic region. While some make appointments prior to discharge, the majority simply recommend follow-up with a particular specialty and provide a list of specified physicians [9]. Addressing variations in discharge planning is important, as patients with inadequate planning are less likely to follow up with their PCP or specialists, which in turn increases the likelihood of readmission. Avoidable ED returns and hospital admissions/readmissions contribute to increased strain on the healthcare system.

Despite the importance of post-ED follow-up, there is considerable variability in patient post-discharge appointment adherence [9]. Risk factors conferring vulnerability for not following up or for having a repeat ED visit within a few days after discharge include low socioeconomic status, male sex, homelessness, arrival to the ED by ambulance, lack of insurance [10,11], and greater age (particularly over 65 years) [12].

Magnusson et al. reviewed the charts of 587 discharged patients and found that 46% of patients who were given a clinic phone number and instructions to call for an appointment (i.e., the standard discharge follow-up process) called for an appointment [13]. However, there was a marked and statistically significant disparity in adherence rates between patients given a specific clinic appointment (65%) vs. the standard process (46%), even controlling for age, the absence of insurance, and no ED consultation with a follow-up physician. Having patient schedule their own follow-up was the factor most associated with poor adherence (p <0.001). This corroborated a previous finding that the patient leaving the ED with only a phone number to call for follow-up rather than with an appointment was the most significant independent correlate of missing follow-up appointments (OR = 3.8) [14].

Consequently, a new model is needed to address the additional challenges faced by vulnerable patients [15]. While it would be ideal to reach out to PCPs and specialists for all patients discharged from the ED, the volume and acuity of an urban ED often limit the ability to do so. Scheduling follow-up appointments prior to the patient’s departure from the ED is associated with increased appointment adherence [7]. In an improved conceptual framework for the ED discharge process, an Emergency Department Discharge Center (EDDC) staffed by ED Care Coordinators would assist patients to obtain post-discharge appointments and track referred patients to determine whether the appointments were attended (and, if not attended, identify and address barriers) [16,17].

Below, we describe the Northwell Health Long Island Jewish Medical Center’s ED Discharge Center from its inception in July 2020 through the end of July 2021. This may assist other EDs in planning and implementing a similar program.

Objectives

The objectives of this study are as follows: (1) describe the structure and activities of the Long Island Jewish Medical Center’s Emergency Department Discharge Center (LIJMC EDDC); (2) characterize the LIJMC EDDC patient population; (3) delineate the volume and specialties of appointments scheduled by the LIJMC EDDC Care Coordinators; (4) demonstrate the impact of the EDDC on LIJMC operational metrics: 72-hour returns, 30-day admissions, and length-of-stay (LOS); (5) evaluate the impact of the EDDC on patient satisfaction.

## Materials and methods

Methods

Data from two sources were collected for the EDDC pilot year (July 1 to July 31, 2021): (1) an MS Excel (Microsoft Corp.; Redmond, WA) database maintained by EDDC Care Coordinators; and (2) data downloaded from the Allscripts "Sunrise"® electronic health record (EHR) by the Emergency Medicine Service Line (EMSL), which included information such as whether the patient had a 72-hour ED re-visit or a 30-day hospital admission within Northwell. The EDDC pilot cohort was compared to non-EDDC "treat-and-release" patients seen in the LIJMC ED during the same period. Certain data (e.g., distance from the patient’s address to the appointment) were only obtainable with certainty from chart review. We conducted a review of a random sample of 139 LIJMC EDDC patients. Statistical significance was determined a priori at p<0.05.

This study was deemed exempt by the Northwell Health Human Subject Protection Program - Institutional Review Board as a quality improvement project.

Implementation of discharge center conceptual framework

Northwell Health Long Island Jewish Medical Center is a 583-bed hospital serving a racially and ethnically diverse population that includes many uninsured or underinsured patients. The typical discharge process for LIJ ED's "non-EDDC" patients consists of providers explaining the patient's confirmed or suspected condition and reasons to return to the ED (i.e., "return precautions"); providing a printout of lab and imaging results; and giving a handout list of relevant follow-up specialists' or PCPs' names and contact information. Patients are responsible for reaching out to those physicians or to physicians with whom they have an existing relationship.

The EDDC, a novel patient care intervention, was developed in July 2020, in the midst of the COVID-19 pandemic. The goals of this innovative program were to: (a) improve patient follow-up after discharge from the ED by having Care Coordinators schedule patient follow-up appointments prior to discharge; (b) decrease health disparities in vulnerable patient populations by facilitating post-discharge care.

Infrastructure

**Figure 1:**
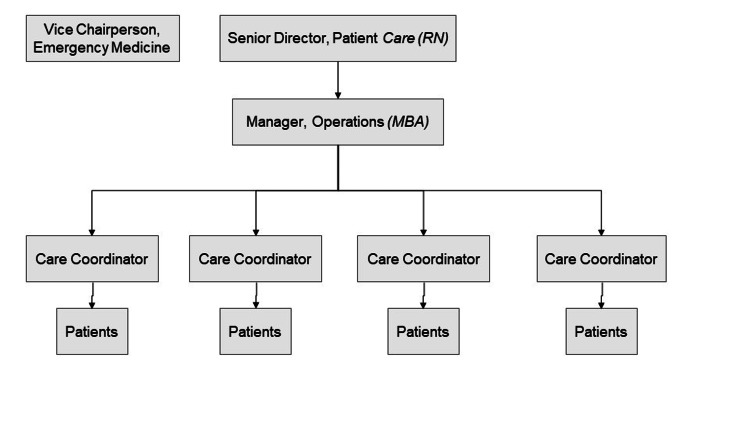
LIJMC EDDC support personnel

The LIJMC EDDC operates Monday-Friday, 7 AM-5 PM, and occasionally on Saturdays. It is housed in a quiet area within the main ED. The center is managed by four care coordinators (minimum education: a high school diploma) under the direction of nursing administration (Figure 1), with input from the Physician Vice Chairperson of Emergency Medicine. Care coordinators carry out a variety of duties to ensure that patients referred to the EDDC are scheduled for timely PCP and/or specialist appointments.

Activities

LIJMC EDDC Care Coordinators require the following information to make an appointment. They obtain this information either by directly interviewing the patient or from the information provided by the ED provider making a referral to the EDDC. (i) The type of specialty and/or PCP follow-up care that is required; (ii) a patient’s diagnosis for which they require follow-up care; (iii) the recommended follow-up time frame; (iv) the patient’s insurance type and coverage status; (v) the patient’s contact information.

Activities of the emergency department provider: There were no guidelines or restrictions on the characteristics of patients (e.g., age, insurance, diagnoses) who could be referred to the EDDC. At any point during or after an ED visit, an ED provider could refer a patient to the discharge center in a multitude of ways: (i) walking to the EDDC and asking care coordinators to visit the patient in the ED at the bedside; (ii) calling the EDDC and asking a care coordinator to visit the patient in the ED at the bedside; (iii) walking the patient to the EDDC; (iv) indicating via the "Sunrise®" EHR "ED Provider Note" (disposition section) that the "patient requires follow-up"; (v) emailing the EDDC to notify the care coordinators that the patient requires follow-up and include the aforementioned information required for scheduling an appointment.

ED Care Coordinators act as patient liaisons by calling and/or emailing physicians’ offices to facilitate appointment scheduling on behalf of the patient. They also work with the patient to select a preferred appointment date, time, and location. The chosen appointment, therefore, is made in accordance with patient availability (when possible) and not necessarily the nearest appointment location or next-available date. Referral appointments are limited to in-system Northwell locations as there is no community-wide database of providers with whom ED Care Coordinators can easily schedule and track appointments. ED Care Coordinators do not have the ability to make appointments for patients who have neither been seen in the ED nor made appointments for patients discharged to a transitional care facility (e.g., a nursing home). Patients whose post-discharge care needs were COVID-related (e.g., infectious disease or pulmonary follow-up; pulse oximeter; outpatient monoclonal antibodies) were coordinated by a separate, non-EDDC, COVID-specific program.

Data Collection

**Figure 2:**
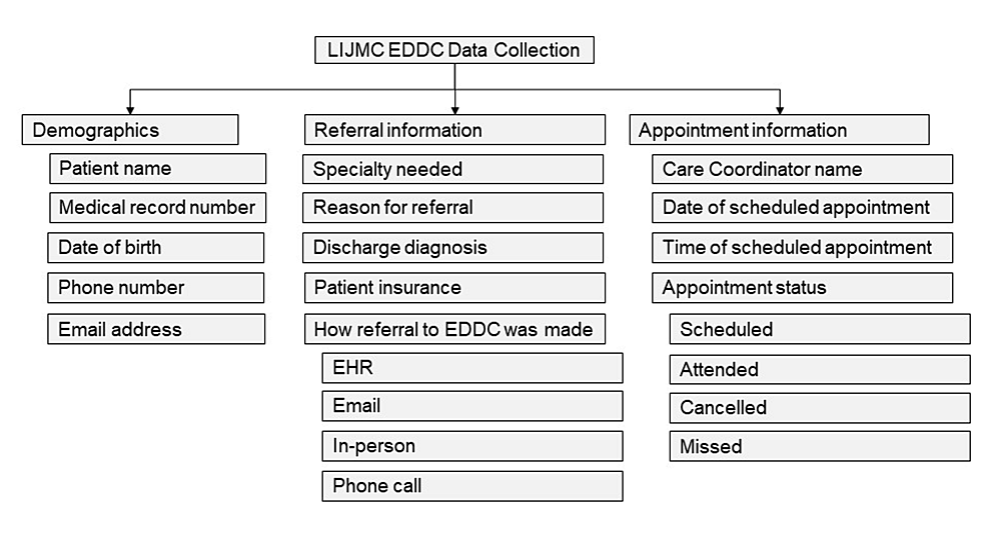
LIJMC EDDC data collection LIJMC: Long Island Jewish Medical Center, EDDC: Emergency Department Discharge Center

Data are obtained for every patient for whom the EDDC is consulted. The collected data points are managed within a HIPAA-compliant file. Data for each patient are collected within the following domains (Figure 2).

Quality Metrics

ED returns within 72 hours and hospital admissions within 30 days have been used as measures to capture the quality of the discharge process and may not be reimbursed by insurance companies. Patient satisfaction is gauged using the Press Ganey® survey question "Likelihood to recommend," a commonly used assessment of patient experience/satisfaction with an office, ED, hospital, or ambulatory surgical facility. We evaluated the impact of the EDDC on these three metrics, in addition to the ED length of stay (LOS).

## Results

During LIJMC’s EDDC pilot year (July 1 to July 31, 2021), 3,616 patients were referred to and managed by the EDDC (average = ~289 patients/month), representing 6.4% of the 56,591 patients discharged from the ED during this timeframe. The characteristics of patients referred to the EDDC are shown in Table 1.

**Table 1:** Characteristics of EDDC patient population (July 2020 to July 2021)

	EDDC patients (3,616)	Non-EDDC patients (52,975)	P-value
Emergency Severity Index Level (1 = most-acute, 5 = least-acute)			
2	8.8%	N/A	N/A
3	75.2%	N/A	N/A
4	15.3%	N/A	N/A
5	0.7%	N/A	N/A
Age (average)	45.7	44.3	<0.0001
Portion of the population ≥65	15.5%	15.4%	0.872
Gender			
Male	44.1%	56.5%	<0.0001
Female	55.9%	43.5%	
Insurance			
Medicaid	31.9%	27.0%	<0.0001
Medicare	11.8%	15.1%	<0.0001
Other/private	51.2%	50.6%	0.485
Uninsured/Self-pay	5.2%	7.4%	<0.0001
Portion of the population with no primary care provider	48.9%	N/A	N/A
Comorbidities			
HTN	5.9%	4.2%	<0.0001
DM	3.7%	2.0%	<0.0001
CHF	0.1%	0.0%	<0.0001
Multiple Comorbidities (2 or more)	7.7%	5.2%	<0.0001
Charlson Comorbidity Index (>5)	9.2%	8.3%	0.0585
Race			
African-American/Black	36.1%	36.1%	1.00
Asian/Hawaiian	22.1%	19.6%	0.0003
Latino	1.2%	1.3%	0.6066
Native American	0.7%	0.8%	0.512
Multi-racial	24.4%	21.3%	<0.0001
White	14.3%	20.4%	<0.0001
Ethnicity			
Hispanic/Latino	16.7%	22.3%	<0.0001
Not Hispanic/Latino	83.3%	77.7%	<0.0001
Portion of the population with non-English speakers	15.8%	Not available	Not available

Although being male is a risk factor for poor post-ED visit follow-up adherence, a smaller percentage of EDDC patients were male. However, this is because a smaller number (26,414 vs. 31,977) and percentage (45.2% vs. 54.8%) of overall ED patients were males. When viewed within gender, an equal percentage of males and females (6.5% vs. 6.3%) were referred to the EDDC (p = 0.7255).

EDDC staff called patients for whom they made an appointment to determine follow-up rates. Of patients referred to the EDDC, 80.0% were able to be contacted, and 59.4% of those (41.7% of all patients referred to the EDDC) had an appointment made. Approximately 70% of patients who had an appointment made attended their appointment (33.4% of all patients referred to the EDDC). Data regarding non-EDDC patient follow-up was unavailable, as it is tracked in a separate ambulatory care EHR, to which EDDC staff and ED providers do not have access.

**Figure 3:**
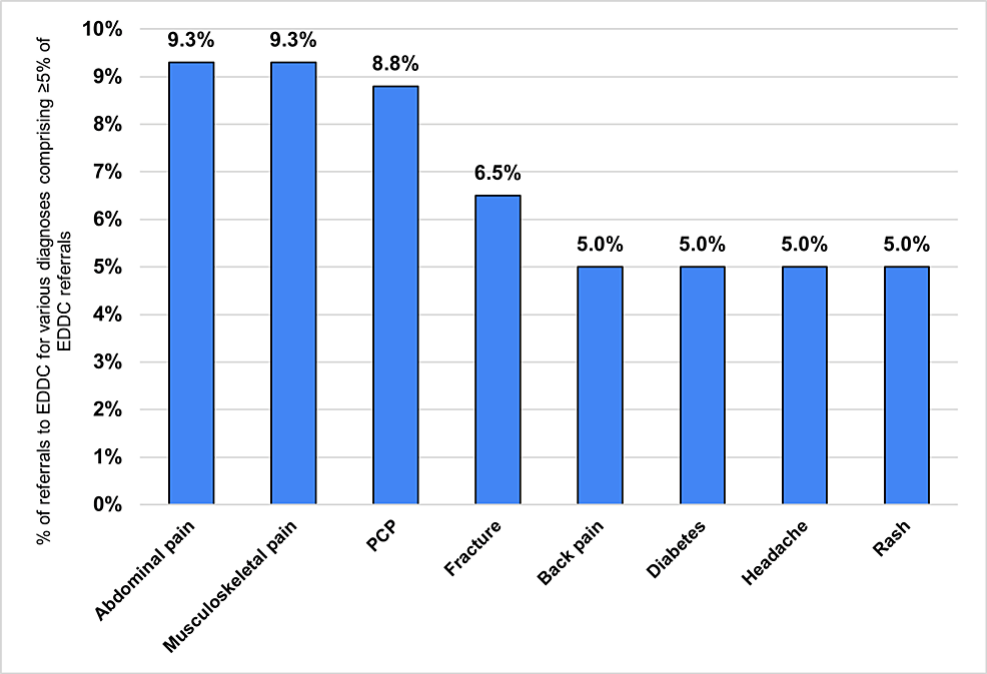
Common diagnoses for which patients were referred to the EDDC

**Figure 4:**
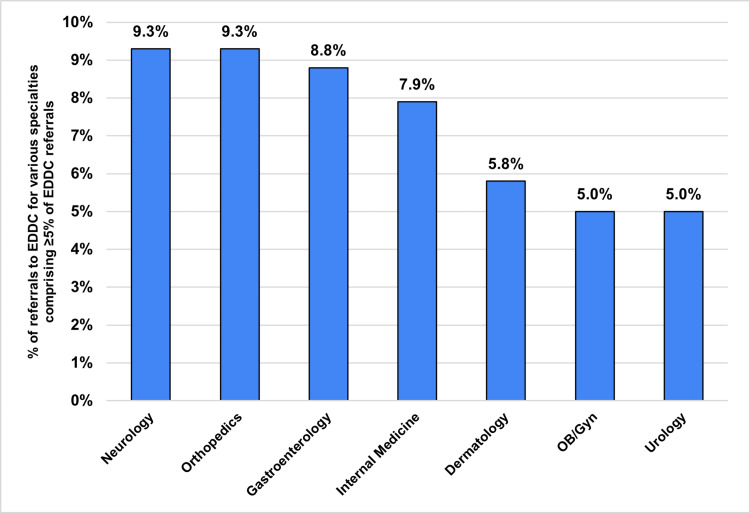
Common specialties requested for EDDC patients

Figure 3 shows the diagnoses comprising at least 5% of the referrals to the EDDC, and Figure 4 shows the specialties to which at least 5% of EDDC patients were referred. "Musculoskeletal" (e.g., back pain, fractures, joint pain) conditions and specialties were the most commonly represented (~20% of conditions and ~15% of specialties). Approximately 10% of referrals were to help patients obtain a PCP. Nearly 90% of referrals were to providers or specialties with whom the patient had not previously established care, rather than re-establishing care with an existing specialty or moving an existing appointment sooner. Scheduled appointments occurred within one week for 54.1% of patients. The majority of referrals (~70%) to the EDDC were made in person (not through email, the EHR, or by phone), despite the restriction on EDDC hours.

Among EDDC patients, distance to appointment (Table 2) was calculated from the patient’s residence zip code to the appointment location zip code, choosing the shortest driving route displayed in Google Maps® (Alphabet, 2021). The median distance between the patient’s residence zip code and the appointment zip code was 8.0 miles, with 13.7% within 5 miles, 57.3% between 5 and 10 miles, and 29.0% greater than 10 miles. There was no statistically significant difference in attendance rates between patients whose distance to the appointment was <5 miles vs. >5 miles. We were unable to obtain such data or perform such analyses for non-EDDC patients.

**Table 2:** Association between appointment attendance rate and distance from home zip code to appointment zip code

Distance from home zip code to appointment zip code	% of all patients	Attended	Missed	P-value compared with <5 miles
<5 miles	13.7%	83.3%	16.7%	1.0
5–10 miles	57.3%	62.7%	37.3%	0.7605
>10 miles	29.0%	78.9%	21.1%	0.7015
Any distance	100.0%	70.2%	29.8%	0.8453

In an unadjusted analysis, EDDC patients were slightly less likely to return to the ED within 72 hours (5.3% vs. 6.5%; p = 0.0044; absolute difference = −1.2%, NNT = 83) or to be hospitalized within 30 days (3.4% vs. 4.2%; p = 0.033; absolute difference = −0.7%, NNT = 143) when compared to "treat-and-release" patients not seen in the EDDC. Even among patients whose scheduled appointment was within 72 hours, there was no statistically significant difference in 72-hour returns (i.e., having an appointment within three days did not prevent an ED re-visit within three days); 2.3% vs. 1.1%, p = 0.5954). EDDC patients had a slightly longer ED LOS (268 vs. 259 minutes, p<0.00001); however, this is unlikely to be operationally significant since patients referred to the EDDC primarily had their interview with care coordinators after completing their ED visit and, having been discharged, no longer necessitated an ED bed. There was no significant difference in satisfaction with the ED visit (2.8% vs. 2.4% would "definitely or probably recommend''; absolute difference = 0.4%; p = 0.1304) (Table 3).

**Table 3:** Comparison of operational metrics between EDDC and non-EDDC patients

	EDDC	non-EDDC	P-value
ED length of stay	268 minutes	259 minutes	<0.00001
Percentage with 72-hour return to ED			
Total population	5.3%	6.5%	0.0044
Patients without insurance	5.9%%	2.4%%	p = 0.003
Patients ≥65	5.3%%	6.1%%	p = 0.4416
Patients without a PCP	6.0%%	7.4%%	p = 0.0647
Percentage with 30-day admission			
Total population	3.4%	4.2%	0.0196
Patients without insurance	4.30%	1.00%	p < 0.0001
Patients ≥65	5.70%	9.20%	p = 0.005
Patients without a PCP	3.40%	3.80%	p = 0.471
Patient satisfaction with ED visit (percent of patients reporting “definitely or probably recommend ED”)	2.8%	2.4%	0.1304

We investigated the impact of the EDDC on specific subsets of vulnerable patients’ short-term ED returns or hospital admissions. Of patients without insurance (i.e., self-pay), 5.9% seen in the EDDC had a 72-hour return vs. 2.4% of non-EDDC patients (p = 0.003), and 4.3% had a 30-day admission vs. 1.0% of non-EDDC patients (p = <0.0001). Of patients 65 years of age or older, 5.3% of EDDC patients vs. 6.1% of non-EDDC patients (p = 0.4416) had a 72-hour return, and 5.7% of EDDC patients had a 30-day admission vs. 9.2% of non-EDDC patients (p = 0.005). Of patients without a PCP, 6.0% of EDDC patients vs. 7.4% of non-EDDC patients (p = 0.0647) had a 72-hour return, and 3.4% of EDDC patients vs. 3.8% of non-EDDC patients (p = 0.471) had a 30-day admission (Table 3).

The majority of patients who returned to the ED within 72 hours or were admitted within 30 days did so with the same chief complaint as their index visit, regardless of whether they had been referred to the EDDC (Table 4).

**Table 4:** Percent of 72-hour returns or 30-day admissions for same chief complaint as index visit

	EDDC	Non-EDDC	P-value
72-hour return	85.7	88.2	0.6006
30-day admission	79.7	82.1	0.7613

Providers were queried regarding their criteria for selecting patients whom they referred to the EDDC. There was great variation in their selection criteria. Stated criteria included: everyone who needs a PCP or specialist; those who require urgent follow-up (e.g., within one to two weeks); those with poor health literacy or for whom English is not their primary language; those who already had difficulty making an appointment; and those whose decision to admit instead of discharge would be influenced by the likelihood of getting a timely outpatient appointment.

## Discussion

Since its inception in July 2020, LIJMC’s EDDC has facilitated the scheduling of over 3,500 appointments with specialists or primary care providers. Patients referred to the EDDC had a modest, but statistically significant, improvement in quality metrics (72-hour ED return visits and 30-day hospital admissions) compared to those referred for outpatient follow-up through standard discharge instructions (non-EDDC patients). These improvements may be largely influenced by the EDDC's having benefited, in particular, the uninsured and elderly. This benefit was identified through univariate, not multivariate, analysis. Future evaluations using multivariate analysis may elucidate this issue. Over half the patients referred to the EDDC had appointments within one week. Patient and provider availability or preferences may have contributed to later appointments. The 10-minute difference in ED LOS, while statistically significant, was not operationally significant, as it did not have a significant impact on the flow of the department since care coordinators saw the majority of patients outside of clinic care areas. While a smaller percentage of patients referred to the DCL were male (44.1%), there was no difference in the percentage of males vs. females that were referred to the DCL. The reason a greater percentage of DCL patients were female is that there were more female patients in the total pool of patients. Anecdotally, providers report satisfaction with the EDDC.

The modest but statistically significant higher rates of 72-hour ED re-visits and 30-day admissions among non-EDDC patients may have been influenced by the greater proportion of such patients having Medicare or being uninsured. Previous studies have identified these as independent risks for discharge failures [11]. While the EDDC and non-EDDC patient populations had a similar average age (~45 years) and proportion ≥65 years, the fact that a higher proportion of non-EDDC patients qualified for Medicare or were uninsured (despite being working-age) suggests they were ill or disabled in ways the EHR may not have captured. Such hidden, confounding differences might explain higher rates of return ED visits and admissions.

The net "effectiveness" of the EDDC (defined as # of patients referred to the EDDC/# of appointments attended) was 33.4%. Patients for whom an appointment was made attended those appointments at rates (~70%) comparable to national clinic show rates, which range from ~70% to 85% (i.e., no-show rates are ~15-30%) [18]. The low overall efficiency reflects that only 80% of patients referred to the EDDC were able to be contacted (many were referred after-hours and so had to be called post-visit) and that, among those who were contacted, an appointment was made for only ~60%. Per EDDC staff, reasons for failure for an appointment to be made include the patient’s insurance not being accepted by Northwell outpatient providers, the patient's preference not to follow up with a Northwell provider, a referral by the patient’s PCP to a different specialist, or an improvement in the patient’s condition.

The median number of miles (7.7) between a patient's zip code and a provider's zip code was shorter than the average national distance (13.8 miles), though this might be because LIJMC is in an urban area. While we did not calculate the average commute time between the patient's home or zip code and the provider's office address or zip code, the estimated commute time would likely be within the range of the national average (34 minutes). It was not possible to obtain the distance between the home zip code and the appointment zip code for non-EDDC patients; hence, a similar analysis was not performed on this group.

Limitations

In this analysis, we were only able to describe the referral and attendance patterns for patients referred to EDDC but not for non-EDDC appointments. The reason for this limitation is that the software for scheduling and tracking appointment status was beyond the purview of the EMSL. Additionally, we were unable to access complete datasets from the EMSL or the Care Coordinators’ Microsoft Excel data table containing certain information deemed pertinent for this analysis (e.g., physicians’ office zip code). Data are currently only electronically abstractable for patients who have scheduled appointments. Consequently, a random sample was used to examine these variables. Data in future analyses will be more comprehensive owing to a new, standardized way of inputting data and pulling data directly from the EHR and scheduling software to automatically track appointment status. In addition, there were no standard guidelines for the types of patients to be referred to the EDDC based on who might most benefit from EDDC services (e.g., non-English-speaking, uninsured) or for how the referral to the EDDC should be made (e.g., in person). These issues will be addressed as the program continues to develop. A limitation of the analysis of "distance to appointment" by using Google Maps® and a zip code rather than the patient's actual address is that the suggested route may not represent the patient’s true commute (distance, method of travel, and associated time) to the appointment. However, the preferred site was chosen in accordance with patient preference, when possible; the patient presumably would have chosen a location where they could arrive expediently. In addition, we were unable to assess 72-hour ED return visits or 30-day admissions for patients who may have attended a hospital other than LIJMC; however, such a limitation applied to both EDDC and non-EDDC patients, and given the overall similarities between the two groups, it is unlikely to represent a threat to the conclusions regarding the EDDC’s effect on these outcomes. Because this pilot program began during the first (peak) year of the COVID pandemic (when people were avoiding ED [19] and outpatient [20] visits), overall ED volume (and referrals to the EDDC and from the EDDC to PCPs and specialists) may have been lower than they otherwise would have been. In addition, our analysis was univariate, not multivariate, and did not adjust for confounding variables that might have influenced the observed differences in 72-hour revisits and 30-day admissions seen in univariate analysis of such variables as age, insurance, and absence of a PCP. This study followed patients for only 30 days and focused on rates of return ED visits and inpatient admissions, whereas the benefits of attending post-ED appointments might extend beyond 30 days and include non-healthcare benefits such as decreased work absenteeism, slower disease progression, improvement in function/ability to perform activities of daily living (ADLs), etc. Finally, only ~6.5% of total ED treat-and-release patients were referred to the EDDC, limiting the power of the study to identify differences between the EDDC and non-EDDC populations.

Future directions

This pilot project was initiated during the first year of the COVID pandemic, a time when ED volume decreased substantially. We expect ED volume to return to its pre-pandemic levels post-pandemic, and EDDC referrals should increase commensurately. As people re-engage with outside activities (e.g., work, recreation), there may be an increase in injuries (e.g., falls, motor vehicle collisions), leading to more orthopedic and podiatric referrals. In addition, we might expect to see more referrals to pulmonology for long COVID.

We intend to implement a standardized screening tool (e.g., the SHOUT tool [11]) as an objective way to assess for vulnerability for discharge failure (i.e., ED return visits) and identify patients who would benefit from the EDDC's services. Greater selectivity will allow care coordinators not only to make appointments for vulnerable patients but also to facilitate attendance by assisting in arranging transportation, child/elder care, etc. Further studies regarding the EDDC may focus on extending the timeframe to look at benefits beyond 30 days and in domains other than healthcare utilization (e.g., work attendance, ADLs).

## Conclusions

The novel LIJMC EDDC program, whose goal is to reduce hurdles to ongoing care for discharged ED patients, produced a modest but statistically significant difference in 72-hour returns and 30-day admissions for patients with EDDC-scheduled appointments when compared with patients referred to outpatient providers using the standard discharge process. ED length of stay increased by 10 minutes for EDDC vs. non-EDDC patients, without a notable difference in ED flow or satisfaction. This increased LOS is unlikely to be operationally significant, as care coordinators assisted most EDDC patients in a non-patient care area. Future analyses will investigate impacts on 72-hour returns, 30-day admissions, LOS, and satisfaction after adjusting for characteristics such as age, insurance, having a PCP, and whether the scheduled appointment was attended.
